# Improved Isolation of Uncultured Anaerobic Bacteria using Medium Prepared with Separate Sterilization of Agar and Phosphate

**DOI:** 10.1264/jsme2.ME19060

**Published:** 2020-02-01

**Authors:** Souichiro Kato, Mia Terashima, Ayano Yama, Megumi Sato, Wataru Kitagawa, Kosei Kawasaki, Yoichi Kamagata

**Affiliations:** 1 Bioproduction Research Institute, National Institute of Advanced Industrial Science and Technology (AIST), 2–17–2–1 Tsukisamu-Higashi, Toyohira-ku, Sapporo 062–8517, Japan; 2 Division of Applied Bioscience, Graduate School of Agriculture, Hokkaido University, Kita-9 Nishi-9, Kita-ku, Sapporo 060–8589, Japan; 3 Institute of Low Temperature Science, Hokkaido University, Kita-19 Nishi-8, Kita-ku, Sapporo, Hokkaido 060–0819, Japan; 4 Hokkaido High-Technology College, 2–12–1 Megumino-kita, Eniwa, Hokkaido 061–1374, Japan; 5 Computational Bio Big Data Open Innovation Laboratory (CBBD-OIL), AIST, 2–17–2–1 Tsukisamu-Higashi, Toyohira-ku, Sapporo 062–8517, Japan; 6 Bioproduction Research Institute, AIST, 1–1–1 Higashi, Tsukuba 305–8567, Japan

**Keywords:** culturability, anaerobic bacteria, wastewater treatment plant, agar medium preparation, phosphate

## Abstract

We previously demonstrated that a simple modification in the preparation of agar media, *i.e.*, autoclaving phosphate and agar separately (termed the “PS protocol”), improved the culturability of aerobic microorganisms by reducing the generation of reactive oxygen species. We herein investigated the effects of the PS protocol on the cultivation of anaerobic microorganisms using sludge from a wastewater treatment system as a microbial source. The application of the PS protocol increased colony numbers and the frequency of phylogenetically novel isolates under aerobic, nitrate reduction, and fermentation conditions. The PS protocol is useful for isolating both aerobic and anaerobic microorganisms.

Only a small portion of the microorganisms present in natural environments may be cultured on agar plate media; this phenomenon is termed the “great plate count anomaly” (Staley and Konopka, 1985; Amann *et al.*, 1995). The recent development of environmental DNA/RNA analysis methods, such as metagenomics, metatranscriptomics, and single cell genomics, has made it possible to estimate the ecophysiology of uncultured microorganisms. However, microbial functions often cannot be deduced from genetic information alone, and the isolation of uncultured microorganisms remains a powerful approach. A number of strategies have been developed to culture fastidious and phylogenetically novel microorganisms (Alain and Querellou, 2009; Vartoukian *et al.*, 2010; Pham and Kim, 2012; Puspita *et al.*, 2012; Epstein, 2013). These include the use of culture platforms that mimic environmental conditions (Kaeberlein *et al.*, 2002; Ferrari *et al.*, 2008), alterations in gelling agents (Tamaki *et al.*, 2009), the addition of antioxidants (Martin *et al.*, 1976) and signal compounds (Bruns *et al.*, 2002), and the physicochemical separation of cells to decrease the negative effects of competitors and inhibitors (Connon and Giovannoni, 2002; Zengler *et al.*, 2002).

We recently demonstrated that a hidden pitfall in the preparation of agar media inhibits colony formation by environmental microorganisms (Tanaka *et al.*, 2014; Kawasaki and Kamagata, 2017). The reactive oxygen species (ROS) generated when agar and phosphate are autoclaved together (termed the “PT” protocol, where “P” is phosphate and “T” indicates “together”) inhibit the growth of some microbes. We demonstrated that the separate sterilization of agar and phosphate (termed the “PS” protocol, where “S” indicates “separate”) minimized ROS generation, which improved the culturability of environmental microorganisms. The PS protocol has been shown to improve the culturability of a wide range of microorganisms, including slow-growing heterotrophs (Kato *et al.*, 2018), hard-to-culture *Actinobacteria* (Adam *et al.*, 2018), and alkane-degrading bacteria (Zheng *et al.*, 2018). Furthermore, many research groups reported the isolation of novel bacterial taxa using the PS protocol (Nishioka *et al.*, 2016; Huang *et al.*, 2017; Kitzinger *et al.*, 2018; Rilling *et al.*, 2018). However, these studies only targeted aerobic microorganisms. Since anaerobic microorganisms (particularly obligate anaerobes) are generally more sensitive to oxidative stress, the PS protocol may be even more effective for the isolation of anaerobic microorganisms. We herein investigated whether the PS protocol promotes colony formation by anaerobic microorganisms and increases the likelihood of isolating phylogenetically novel microorganisms.

Two different anaerobic conditions (nitrate reduction and fermentation conditions) and an aerobic condition as a control were examined to evaluate the effects of the PS protocol on the culturability of microorganisms. The agar plates used to isolate aerobic and anaerobic microorganisms were prepared as described previously (Tanaka *et al.*, 2014; Kawasaki and Kamagata, 2017). Medium constituents were grouped into three solutions, *i.e.*, basal medium, energy and carbon sources, and phosphate buffer. The basal medium was comprised of (final concentrations) 18.7‍ ‍mM NH_4_Cl, 0.5‍ ‍mM MgCl_2_, 0.1‍ ‍mM MgSO_4_, 0.5‍ ‍mM CaCl_2_, 10.3‍ ‍mM NaCl, 0.1 g L^–1^ of Bacto yeast extract, 15 g L^–1^ of Bacto agar, and 10 mL L^–1^ each of a trace element solution and a vitamin solution (Kato *et al.*, 2016). The energy and carbon source solution contained sodium acetate (final concentration 10‍ ‍mM) for the aerobic culture, sodium acetate plus sodium nitrate (final concentration 10‍ ‍mM each) for the nitrate reduction culture, and Bacto peptone, Bacto yeast extract, and glucose (final concentration 0.2 g L^–1^ each) for the fermentation culture. The phosphate buffer solution (pH 7.2) was comprised of KH_2_PO_4_ and K_2_HPO_4_ (final concentration of 10‍ ‍mM each). In the PT medium, the basal medium and phosphate buffer were mixed before autoclaving, and the filter-sterilized energy and carbon source solution was added separately before the medium was poured. In the PS medium, the three solutions were sterilized separately and subsequently mixed. Sludge from the oxidation ditch plant treating domestic wastewater in Okishima, Omihachiman, Shiga, Japan, was used as the microbial source. This plant is operated with a repetitive cycle of aerobic and anaerobic phases, and has been shown to contain diverse aerobic and both facultative and obligate anaerobic microorganisms (Terashima *et al.*, 2016). The plant operating conditions and sampling procedures were described previously (Terashima *et al.*, 2016). The sludge sample was suspended in sterilized saline (0.9% NaCl) and diluted in a 10-fold series. Aliquots (100 μL) from each dilution were inoculated onto agar media with five replicates and incubated at 25°C in the dark. Anaerobic cultures were conducted using an AnaeroPack pouch bag with an AnaeroPack oxygen absorber (Mitsubishi Gas Chemical). The number of colony-forming units (CFUs) on each agar plate was counted during the incubation. Only plates with 20 to 200 CFUs were included in the cultivation results reported.

To evaluate the effects of the medium preparation protocol (PT vs. PS) on the culturability of aerobic and anaerobic microorganisms, the CFUs obtained on each agar plate under three different culture conditions were compared (Fig. 1). The number of CFUs obtained under aerobic conditions was significantly higher for the PS protocol than for the PT protocol (Fig. 1A), which is consistent with our previous findings (Tanaka *et al.*, 2014; Kawasaki and Kamagata, 2017; Kato *et al.*, 2018). Furthermore, CFU counts were 2- to 3-fold higher on plates prepared using the PS protocol under nitrate reduction and fermentation conditions (Fig. 1B and C). This result suggests that the alleviation of oxidative stress by the PS protocol is also effective for improving the culturability of anaerobic microorganisms.

We conducted a phylogenetic analysis of isolates obtained from each culture condition. Colonies were randomly picked from the agar plates and transferred to fresh agar plates prepared using the corresponding PS or PT protocol for further purification. The partial 16S rRNA gene was amplified by colony PCR using a universal primer set for bacteria, 27F (5′-AGA GTT TGA TCM TGG CTC AG-3′) and 533R (5′-TTA CCG CGG CKG CTG RCA C-3′). PCR products were purified using a QIAquick PCR Purification Kit (QIAGEN) in accordance with the manufacturer’s instructions. PCR products were sequenced by the TaKaRa Bio Company using the 533R primer. The sequences obtained were assigned to phylotypes using the BLASTClust program (Altschul *et al.*, 1997) with a 97% sequence identity cut-off. The phylogenetic classification of each phylotype was performed using the RDP Classifier (Wang *et al.*, 2007). The closest relatives of each phylotype were inferred using the BLAST program (Altschul *et al.*, 1997).

A total of 296 strains (135, 86, and 75 strains from aerobic, nitrate reduction, and fermentation conditions, respectively) were isolated and sequenced. The results of the phylogenetic analysis at the class level are shown in Fig. 2. Isolates from aerobic cultures were classified into the classes of *Alpha-*, *Beta-*, and *Gamma-proteobacteria*, *Actinobacteria*, and *Bacilli*. *Betaproteobacteria* were more abundantly isolated from PS medium, whereas *Actinobacteria* were more abundant in PT isolates. These results are consistent with our previous findings, which were obtained using soil and freshwater sediments as the microbial sources (Tanaka *et al.*, 2014; Kato *et al.*, 2018). The majority of the isolates recovered under anaerobic conditions (nitrate reduction and fermentation conditions) were classified into *Proteobacteria*, *Actinobacteria*, *Bacilli*, and *Clostridia*. Of note, isolates classified into *Clostridia* were only recovered from PS medium. This result is consistent with the majority of *Clostridia* strains being obligate anaerobes that are sensitive to oxidative stress, suggesting that the PS protocol is effective for isolating these strictly anaerobic microorganisms.

To clarify whether modifications to the culture method allowed us to isolate more diverse microorganisms, we calculated the Shannon diversity index (H) for isolates from each culture condition using the number of phylotypes and number of strains assigned to each phylotype (Table 1). Since the PS protocol may not have any factors that may negatively affect microbial growth, we expected the diversity in the isolates from PS medium to be higher than that from PT medium. In our previous studies targeting aerobic microorganisms in soils and freshwater sediments, the diversity indexes of isolates obtained using PS medium were higher than those from PT medium (Tanaka *et al.*, 2014; Kato *et al.*, 2018). In the present study, the isolation of anaerobic microorganisms under fermentation conditions revealed the same results, *i.e.*, more diverse microorganisms were obtained using the PS protocol than the PT protocol (H=2.39 vs. 1.62). However, under the other culture conditions, the diversity index did not markedly differ between PS and PT media (aerobic, H=3.09 vs. 3.26; nitrate reduction, H=2.72 vs. 2.52). Although the reason for this remains unknown, microbes may preferentially and predominantly grow on PS plates, which may decrease diversity.

To clarify whether the PS protocol increased the frequency of isolating phylogenetically novel microorganisms, “novelty indexes” were calculated for each culture condition. Representative sequences from each phylotype were subjected to an RDP Classifier analysis (Wang *et al.*, 2007), and phylotypes with less than 80% classification reliability at the genus level were defined as novel. The novelty index for each condition was calculated as “the number of novel phylotypes/total number of isolates” (Table 1). Similar to our previous studies (Tanaka *et al.*, 2014; Kato *et al.*, 2018), aerobic isolates from PS medium had a higher novelty index than those from PT medium (0.194 vs. 0.074). Furthermore, anaerobic microorganisms showed the same results; PS isolates contained a greater proportion of phylogenetically novel bacteria than PT isolates (nitrate reduction condition, 0.222 vs. 0.122; fermentation condition, 0.100 vs. 0.029). These results suggest that the application of the PS protocol improves the culturability of previously uncultured anaerobic microorganisms.

The anaerobic isolates obtained in the present study included several strains with high phylogenetic novelty that may only be isolated using the PS protocol. The phylotype YS37 (the family *Rhodocyclaceae*, class *Betaproteobacteria*, Table S1), was only isolated from PS medium under fermentation conditions. The closest relative of phylotype YS37 was “*Candidatus* Accumulibacter phosphatis” (94.8% sequence identity). Although “*Ca.* A. phosphatis” is known as a polyphosphate-accumulating bacterium that contributes to the recovery of phosphate in various wastewater treatment systems, it has yet to be isolated in axenic cultures (Yuan *et al.*, 2012). Since bacteria in the family *Rhodocyclaceae* were previously shown to contribute to phosphate removal in the wastewater treatment plant used in the present study (Terashima *et al.*, 2016), the phylotype YS37 may be a previously unidentified bacterium with high phosphate removal activity. Furthermore, the phylotypes YS38 and YS76, which were also isolated only from PS media under nitrate reduction and fermentation conditions, respectively, showed low sequence identities to known isolated species (88.9% identity to *Acidovorax caeni* and 90.5% identity to *Ornithinimicrobium algicola*) (Table S1). Additional investigations on their physiologies (*e.g.*, sensitivity to oxidative stress) will help to clarify why they may be isolated using the PS protocol.

In conclusion, the present study demonstrated that the separate sterilization of phosphate and agar during medium preparation (*i.e.*, the PS protocol) improved the culturability of anaerobic microorganisms and increased the potential for isolating phylogenetically novel microorganisms. Obligate anaerobes (*e.g.*, *Clostridia* strains) were only recovered from PS medium. The application of the PS protocol to diverse anaerobic environmental samples will enable the isolation of phylogenetically and functionally novel anaerobic microorganisms.

## Nucleotide sequence accession numbers

The GenBank/EMBL/DDBJ accession numbers for the 16S rRNA gene sequences of isolates obtained in the present study are LC471496–LC471583.

## Supplementary Material

Supplementary Material

## Figures and Tables

**Fig. 1.**
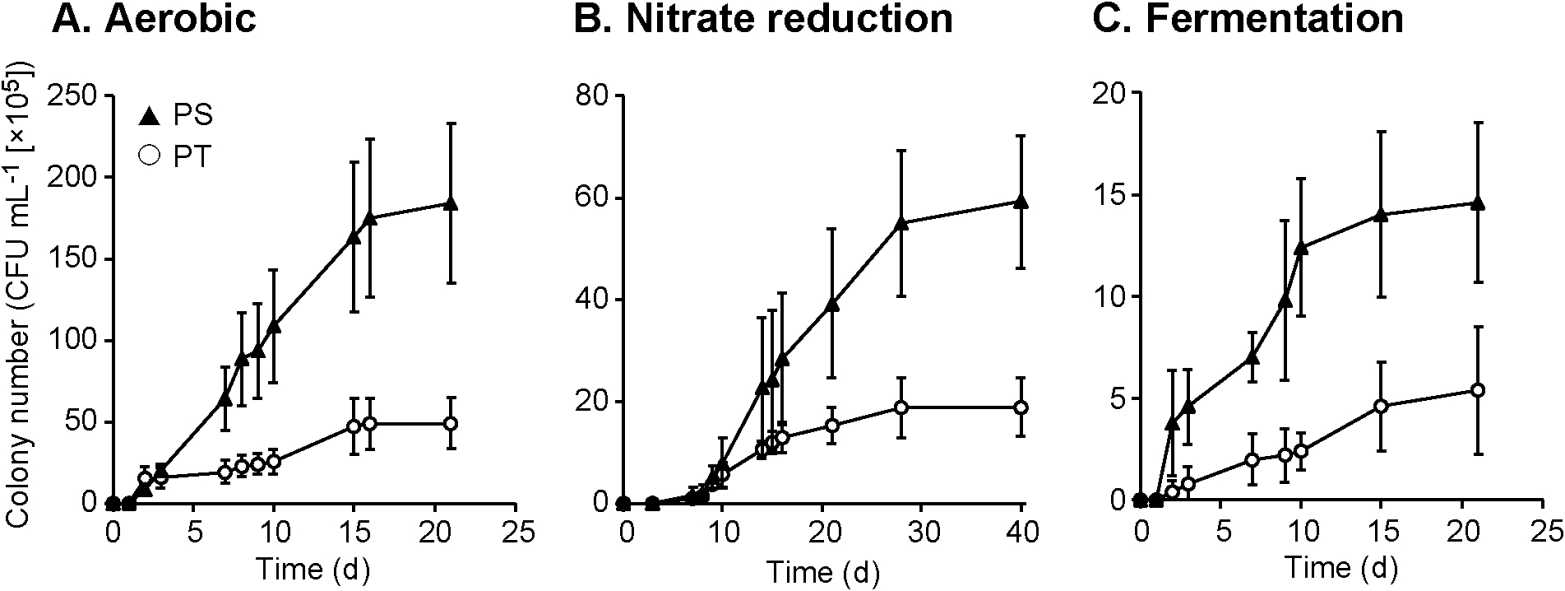
Total colony numbers (colony-forming units, CFUs) obtained from PT (phosphates and agar autoclaved together) and PS (phosphates and agar autoclaved separately) agar media under aerobic (A), nitrate reduction (B), and fermentation (C) conditions. CFU counts are averages from five replicate agar plates. Error bars represent standard deviations.

**Fig. 2.**
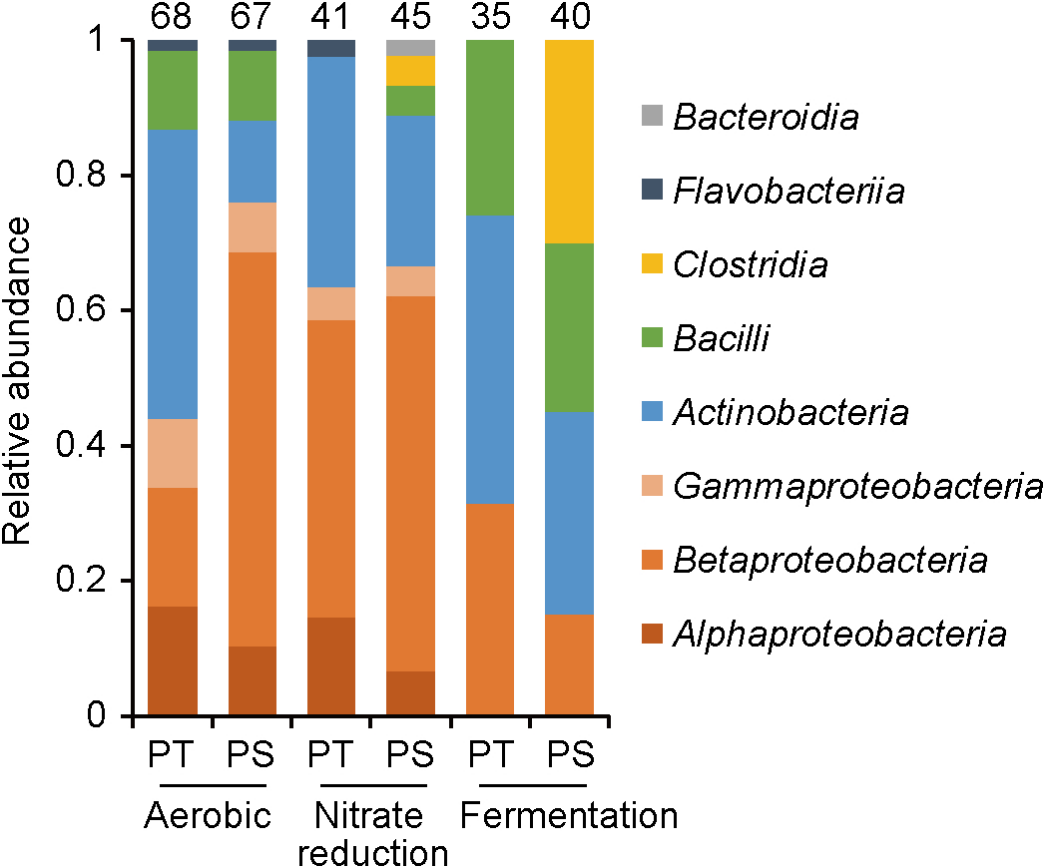
Phylogenetic distribution of strains isolated from a wastewater treatment system using PT (phosphates and agar autoclaved together) and PS (phosphates and agar autoclaved separately) media under aerobic, nitrate reduction, and fermentation conditions. Isolates were classified at the class level using the RDP Classifier based on partial 16S rRNA gene sequences. The number above each bar indicates the number of isolates obtained under each isolation condition.

**Table 1.** Diversity and novelty of microorganisms isolated from wastewater treatment samples using different isolation procedures.

Culture conditions	Number of isolates	Number of phylotypes^a^	Shannon diversity index	Number of novel phylotypes^b^	Novelty index^c^
Aerobic					
PT	68	34	3.26	5	0.074
PS	67	40	3.09	13	0.194
Nitrate reduction					
PT	41	19	2.55	5	0.122
PS	45	24	2.72	10	0.222
Fermentation					
PT	35	6	1.62	1	0.029
PS	40	13	2.39	4	0.100

^a^ Isolates were assigned to phylotypes based on partial 16S rRNA gene sequences, with a sequence identify cut-off value of 97%. ^b^ Phylotypes with less than 80% classification reliability at the genus level in the RDP Classifier analysis were defined as novel. ^c^ The novelty index was calculated as “the number of novel phylotypes/total number of isolates”.
