# Supplementary material for: Improved Isolation of Uncultured Anaerobic Bacteria using Medium Prepared with Separate Sterilization of Agar and Phosphate
Source: Microbes Environ. 2020 Feb 1;35(1):ME19060. doi: 10.1264/jsme2.ME19060 (PMC7104283; doi:10.1264/jsme2.ME19060)
Supplement: Supplementary file 1 — Supplementary Material [file 35_19060_s1.pdf]

Table S1. Phylotypes of isolates. Phylogenetically novel phylotypes (with RDP Classifier confidence values <80%) are shown in bold.

		Number of isolates											
		Aerobic		Nitrate-reducing		Fermenting		Phylogenetic information (RDP classifier)			BLAST analysis		
Phylotype	Accession number	PT	PS	PT	PS	PT	PS	Class	Genus	Confidence	Closest relative	Accession number	Identity, %
YS01	LC471496	4			1			**Alphaproteobacteria**	**Paenirhodobacter**	**0.39**	Sinirhodobacter ferrireducens strain SgZ-3	NR_159103	99.1
YS02	LC471497	2		3				**Alphaproteobacteria**	**Pleomorphomonas**	**0.38**	Pleomorphomonas koreensis strain NBRC 100803	NR_113942	92.5
YS03	LC471498	3						Alphaproteobacteria	Mesorhizobium	0.91	Mesorhizobium tamadayense strain Gsoil318-1	MG461201	99.3
YS04	LC471499			2	1			**Alphaproteobacteria**	**Ochrobactrum**	**0.62**	Ochrobactrum anthropi strain IS1	KM017733	99.8
YS05	LC471500		2					Alphaproteobacteria	Roseomonas	1	Roseomonas lacus strain BF3	M691116	98.0
YS06	LC471501			1	1			**Alphaproteobacteria**	**Azorhizobium**	**0.61**	Xanthobacter flavus strain RS60	MH715209	96.7
YS07	LC471502	1						Alphaproteobacteria	Paenirhodobacter	0.94	Paenirhodobacter enshiensis strain wi-144	MK039093	97.6
YS08	LC471503	1						**Alphaproteobacteria**	**Rhizobium**	**0.34**	Rhizobium puerariae strain PC004	NR_148819	95.8
YS09	LC471504		1					**Alphaproteobacteria**	**Rhodobacter**	**0.38**	Rhodobacter blasticus strain HWS0445	LN835430	92.8
YS10	LC471505		1					**Alphaproteobacteria**	**Defluviimonas**	**0.49**	Pseudorhodobacter sinensis strain Y1R2-4	NR_151911	96.7
YS11	LC471506		1					**Alphaproteobacteria**	**Ancalomicrobium**	**0.46**	Ochrobactrum anthropi strain BGRI-SK10	MF928879	94.3
YS12	LC471507		1					**Alphaproteobacteria**	**Mesorhizobium**	**0.76**	Agrobacterium rhizogenes strain: NGT471	AB289616	99.2
YS13	LC471508		1					Alphaproteobacteria	Novosphingobium	1	Novosphingobium ginsenosidimutans strain FW-6	NR_133800	97.4
YS14	LC471509	3	8	2	4	11	6	Betaproteobacteria	Thauera	1	Thauera aromatica strain LG356	AJ315680	97.0
YS15	LC471510		6	9	9			Betaproteobacteria	Zoogloea	1	Zoogloea caeni strain STM89	KY393074	96.6
YS16	LC471511	4	1	3	7			Betaproteobacteria	Acidovorax	0.98	Acidovorax caeni strain KmW3200907	MG011567	99.8
YS17	LC471512		4		1			**Betaproteobacteria**	**Dechloromonas**	**0.56**	Dechloromonas hortensis strain HYN0024	KY029047	99.3
YS18	LC471513		3					Betaproteobacteria	Thauera	1	Thauera aminoaromatica strain R2	MK271352	100.0
YS19	LC471514		3					**Betaproteobacteria**	**Azovibrio**	**0.25**	Thauera mechernichensis isolate TOSS-142	LN650468	85.8
YS20	LC471515	2						Betaproteobacteria	Vitreoscilla	0.9	Vitreoscilla stercoraria strain Gottingen 1488-6	NR_025894	94.2
YS21	LC471516	2						Betaproteobacteria	Ideonella	0.9	Ideonella dechloratans strain BK-22	KU360710	99.1
YS22	LC471517		2					Betaproteobacteria	Zoogloea	0.96	Zoogloea resiniphila strain MMB	KU321684	95.3
YS23	LC471518		2					Betaproteobacteria	Aquabacterium	0.95	Aquabacterium commune strain IMCC34942	MK226318	98.4
YS24	LC471519			2				Betaproteobacteria	Brachymonas	0.97	Brachymonas denitrificans strain M2/14	KX826979	93.9
YS25	LC471520		1	1				Betaproteobacteria	Uruburuella	1	Uruburuella suis strain A51	HQ259692	99.8
YS26	LC471521			1	1			Betaproteobacteria	Zoogloea	0.9	Zoogloea caeni strain STM89	KY393074	93.7
YS27	LC471522	1						Betaproteobacteria	Comamonas	1	Comamonas terrae strain RBL17-27	MG518393	98.4
YS28	LC471523		1					Betaproteobacteria	Acidovorax	1	Acidovorax temperans isolate OTU-d24	KJ147089	100.0
YS29	LC471524		1					Betaproteobacteria	Zoogloea	1	Zoogloea caeni strain STM89	KY393074	95.6
YS30	LC471525		1					Betaproteobacteria	Hydrogenophaga	1	Hydrogenophaga pseudoflava strain STM41	KY393035	100.0
YS31	LC471526		1					**Betaproteobacteria**	**Zoogloea**	**0.76**	Zoogloea caeni strain STM89	KY393074	93.6
YS32	LC471527		1					**Betaproteobacteria**	**Xylophilus**	**0.46**	Variovorax ginsengisoli strain zw90	MH338003	91.2
YS33	LC471528		1					Betaproteobacteria	Diaphorobacter	0.86	Diaphorobacter oryzae strain 3R2-14	GU300152	100.0
YS34	LC471529		1					Betaproteobacteria	Zoogloea	1	Zoogloea oryzae strain NBRC 102407	NR_114069	99.8
YS35	LC471530		1					**Betaproteobacteria**	**Rivicola**	**0.11**	Thauera aminoaromatica strain R2	MK271352	88.1
YS36	LC471531		1					**Betaproteobacteria**	**Ferribacterium**	**0.77**	Dechloromonas hortensis strain HYN0024	KY029047	85.9
YS37	LC471532				1			**Betaproteobacteria**	**Propionivibrio**	**0.72**	Candidatus Accumulibacter phosphatis clade IIA str. UW-1	CP001715	94.8
YS38	LC471533				1			**Betaproteobacteria**	**Alicycliphilus**	**0.15**	Acidovorax caeni strain KmW3200907	MG011567	88.9
YS39	LC471534				1			Betaproteobacteria	Alicycliphilus	0.8	Alicycliphilus denitrificans strain ADC-14	KM210246	95.4
YS40	LC471535	3		1				Gammaproteobacteria	Klebsiella	0.94	Klebsiella quasipneumoniae subsp. quasipneumoniae stra	MK336743	100.0
YS41	LC471536	3						Gammaproteobacteria	Enterobacter	0.95	Enterobacter cloacae strain XL3-1	MF197498	100.0
YS42	LC471537		2					Gammaproteobacteria	Pseudomonas	1	Pseudomonas alcaligenes strain MnS2201007	MG011587	100.0
YS43	LC471538	1	1					Gammaproteobacteria	Pseudomonas	1	Pseudomonas panipatensis strain B1M30	JN644068	99.5
YS44	LC471539			1	1			Gammaproteobacteria	Tolumonas	1	Tolumonas auensis strain DSM 9187	NR_074805	99.6
YS45	LC471540		1					Gammaproteobacteria	Acinetobacter	1	Acinetobacter tjernbergiae strain DX 14971	KR094129	99.1

YS46	LC471541		1					**Gammaproteobacteria**	**Kluyvera**	**0.48**	Lelliottia amnigena strain Md1-55	MF581461	90.9
YS47	LC471542				1			**Gammaproteobacteria**	**Tolumonas**	**0.62**	Pseudaeromonas pectinilytica strain AR1	NR_156860	98.5
YS48	LC471543			5	2	9	1	**Actinomycetales**	**Brooklawnia**	**0.66**	Brooklawnia cerclae strain BL-34	NR_043631	93.9
YS49	LC471544				3		5	**Actinomycetales**	**Propionicimonas**	**0.68**	Propionicimonas paludicola strain Wd	NR_104769	95.9
YS50	LC471545	4	2					Actinomycetales	Mycobacterium	1	Mycobacterium wolinskyi strain UMBR 0005	KY243958	98.6
YS51	LC471546	4	1					Actinomycetales	Mycobacterium	1	Mycobacterium moriokaense strain: S32433-a	AB649000	99.3
YS52	LC471547			3	2			Actinomycetales	Tessaracoccus	0.99	Tessaracoccus flavescens strain CM1_SD_64	MH174449	99.8
YS53	LC471548	3	1					Actinomycetales	Microbacterium	0.86	Microbacterium lacus strain STM55	KY393060	98.9
YS54	LC471549	2			1			Actinomycetales	Microbacterium	0.96	Microbacterium dextranolyticum strain Na27	HQ831382	99.5
YS55	LC471550	3						Actinomycetales	Gordonia	1	Gordonia sputi strain Z1-2	KJ571101	99.1
YS56	LC471551	2						Actinomycetales	Rhodococcus	1	Rhodococcus qingshengii strain HPJ	MH938043	100.0
YS57	LC471552	2						Actinomycetales	Rhodococcus	1	Rhodococcus jostii strain CR59	MF796707	99.0
YS58	LC471553	1	1					**Actinomycetales**	**Millisia**	**0.2**	Gordonia phthalatica strain QH-11	NR_159081	92.9
YS59	LC471554			2		5	3	Actinomycetales	Propionicimonas	0.94	Propionicimonas paludicola strain Wd	NR_104769	97.7
YS60	LC471555			1	1		1	Actinomycetales	Propioniciclava	1	Propioniciclava tarda strain WR061	NR_112669	95.6
YS61	LC471556			1	1		1	**Actinomycetales**	**Actinotignum**	**0.63**	Myceligenerans xiligouense strain R-31601	AM943043	97.2
YS62	LC471557					1	1	Actinomycetales	Propionibacterium	1	Propionibacterium cyclohexanicum strain JCM 21245	NR_113380	98.6
YS63	LC471558	1						Actinomycetales	Brevibacterium	1	Brevibacterium sanguinis strain wx5	KF963622	100.0
YS64	LC471559	1						**Actinomycetales**	**Skermania**	**0.19**	Gordonia phthalatica strain QH-11	NR_159081	91.4
YS65	LC471560	1						Actinomycetales	Gordonia	0.99	Gordonia phthalatica strain QH-11	NR_159081	95.3
YS66	LC471561	1						Actinomycetales	Rhodococcus	1	Rhodococcus equi strain N107	HM244991	99.5
YS67	LC471562	1						Actinomycetales	Mycobacterium	1	Mycobacterium rhodesiae strain I3_7	KT873847	100.0
YS68	LC471563	1						Actinomycetales	Gordonia	1	Gordonia hongkongensis strain 5-5	MK277458	97.9
YS69	LC471564	1						Actinomycetales	Mycobacterium	0.86	Mycobacterium rhodesiae strain S047	FJ544426	95.0
YS70	LC471565	1						Actinomycetales	Mycobacterium	1	Mycobacterium mageritense strain OS3-93	FN178448	97.4
YS71	LC471566		1					Actinomycetales	Tetrasphaera	0.98	Tetrasphaera remsis strain 3-M5-R-4	NR_104693	98.2
YS72	LC471567		1					Actinomycetales	Micromonospora	0.96	Micromonospora lupini strain CKG1	KF447938	99.1
YS73	LC471568		1					Actinomycetales	Nocardioides	0.95	Nocardioides conyzicola strain HWE 2-02	NR_135730	91.2
YS74	LC471569			1				Actinomycetales	Actinomyces	1	Actinomyces naeslundii strain CCUG 33914	AJ234048	92.8
YS75	LC471570			1				Actinomycetales	Tessaracoccus	1	Tessaracoccus flavescens strain SST-39T	CP019607	99.6
YS76	LC471571						1	**Actinomycetales**	**Aestuariimicrobium**	**0.72**	Ornithinimicrobium algicola strain JC311	NR_145890	90.5
YS77	LC471572	2	3			6	4	Bacilli	Bacillus	1	Bacillus subtilis strain V37	MK229103	99.4
YS78	LC471573	2	2		1	3	5	Bacilli	Bacillus	1	Bacillus mycoides F-39	LC430064	99.8
YS79	LC471574	2	1					Bacilli	Bacillus	1	Bacillus altitudinis strain P10-H1-2	MK318589	100.0
YS80	LC471575	2						Bacilli	Staphylococcus	1	Staphylococcus epidermidis strain B0021-03R	MH447005	100.0
YS81	LC471576		1					**Bacilli**	**Salirhabdus**	**0.32**	Bacillus cereus strain X1	HQ917116	94.3
YS82	LC471577				1			Bacilli	Bacillus	0.92	Bacillus drentensis strain NA-15	KU254659	100.0
YS83	LC471578						1	Bacilli	Trichococcus	1	Trichococcus pasteurii strain H181a	EF204309	99.8
YS84	LC471579				1		9	Clostridia	Clostridium sensu strict	1	Clostridium perfringens strain XJWB01	KX094441	100.0
YS85	LC471580				1		2	Clostridia	Clostridium sensu strict	1	Clostridium butyricum strain Sx-01	MH259843	100.0
YS86	LC471581	1	1					Flavobacteriia	Cloacibacterium	1	Cloacibacterium rupense strain STM31	KY393025	100.0
YS87	LC471582			1				Flavobacteriia	Cloacibacterium	1	Cloacibacterium haliotis strain WB5	NR_125655	99.6
YS88	LC471583				1			Bacteroidia	Macellibacteroides	0.98	Macellibacteroides fermentans strain PC72	MF800883	99.4
Total number		68	67	41	45	35	40						
